# Supplementary material for: A visual–omics foundation model to bridge histopathology with spatial transcriptomics
Source: Nat Methods. Author manuscript; Available in PMC 2025 Jul 25. (PMC12240810; doi:10.1038/s41592-025-02707-1)
Supplement: Supplementary Material [file NIHMS2092004-supplement-Supplementary_Material.pdf]

nature methods

Supplementary information https://doi.org/10.1038/s41592-025-02707-1

A visual–omics foundation model to bridge histopathology with spatial transcriptomics

In the format provided by the authors and unedited

Table of Contents

Supplementary Figures 2

Supplementary Fig. 1 Evaluation of tissue alignment using PCC and Kendall's tau coefficient 2

Supplementary Fig. 2 Examples of tissue alignment 3

Supplementary Fig. 3 Tissue alignment of fine-tuning, pre-training, and train from scratch 4

Supplementary Fig. 4 Tissue alignment using dual-modalities and single-modality 5

Supplementary Fig. 5 Cell type annotation.. 6

Supplementary Fig. 6 H&E image of the brain sample 7

Supplementary Fig. 7 In-house patient samples 8

Supplementary Fig. 8 Loki ST gene expression prediction workflow 9

Supplementary Notes 10

Supplementary Note 1: Evaluating OmiCLIP's transcriptomic encoder on single-cell RNA-seq data 11

Supplementary Note 2: Cancer patient risk stratification in the TCGA dataset using OmiCLIP embeddings 13

Supplementary Note 3: Downstream evaluation datasets 18

Supplementary Notes references 20

Supplementary Figures

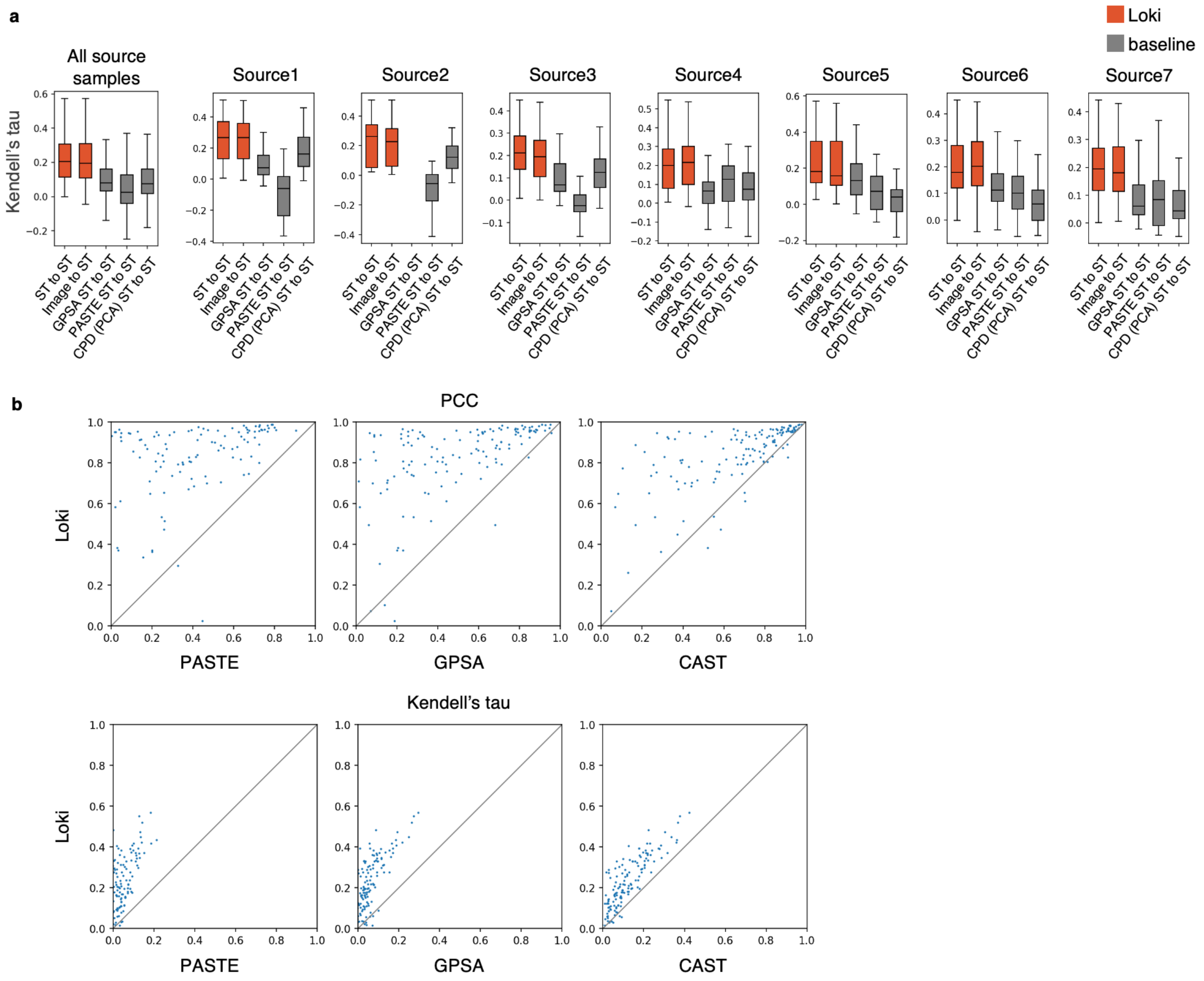

Supplementary Fig. 1: Evaluation of tissue alignment using PCC and Kendall's tau coefficient.

a, Boxplots show the comparison of tissue alignment performances on 7 source samples of 8 adjacent normal human small intestine samples respectively and combined, represented by the Kendall's tau coefficient of highly variable gene expression between target and source sample after alignment at the same location, using Loki and baseline methods (PASTE, GPSA and CPD method) respectively. In the box plots, the middle line represents the median, the box boundaries indicate the interquartile range, and the whiskers extend to data points within 1.5× the interquartile range.

b, Comparison of PCC and Kendall's tau coefficient on 2 adjacent human ovarian carcinosarcoma samples using ST-to-ST tissue alignment between Loki and PASTE, Loki and GPSA, and Loki and CAST, respectively.

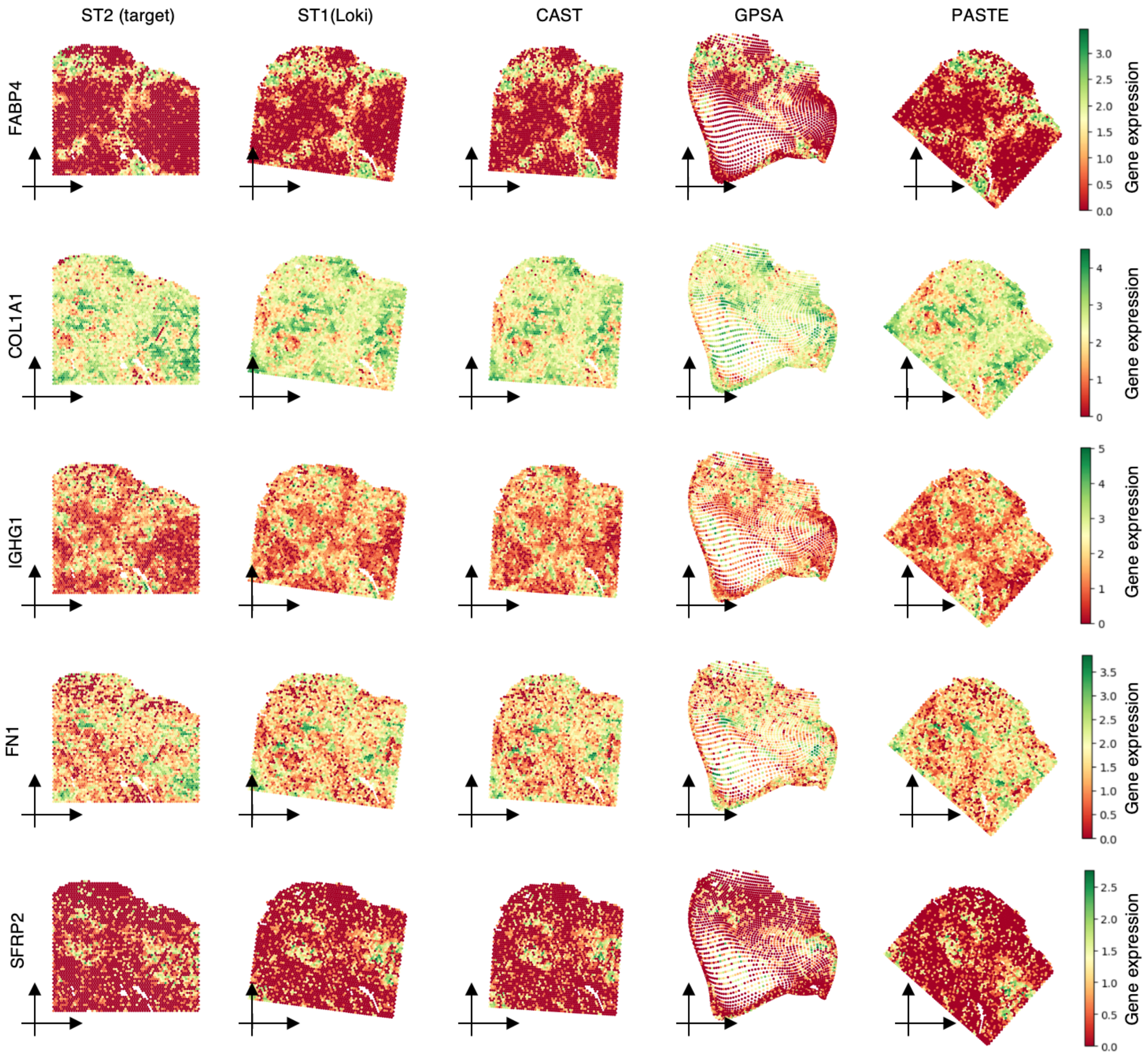

Supplementary Fig. 2: Examples of tissue alignment.

Gene expression of target sample and source sample after alignment using Loki (ST-to-ST), CAST(ST-to-ST), GPSA(ST-to-ST), and PASTE (ST-to-ST), respectively.

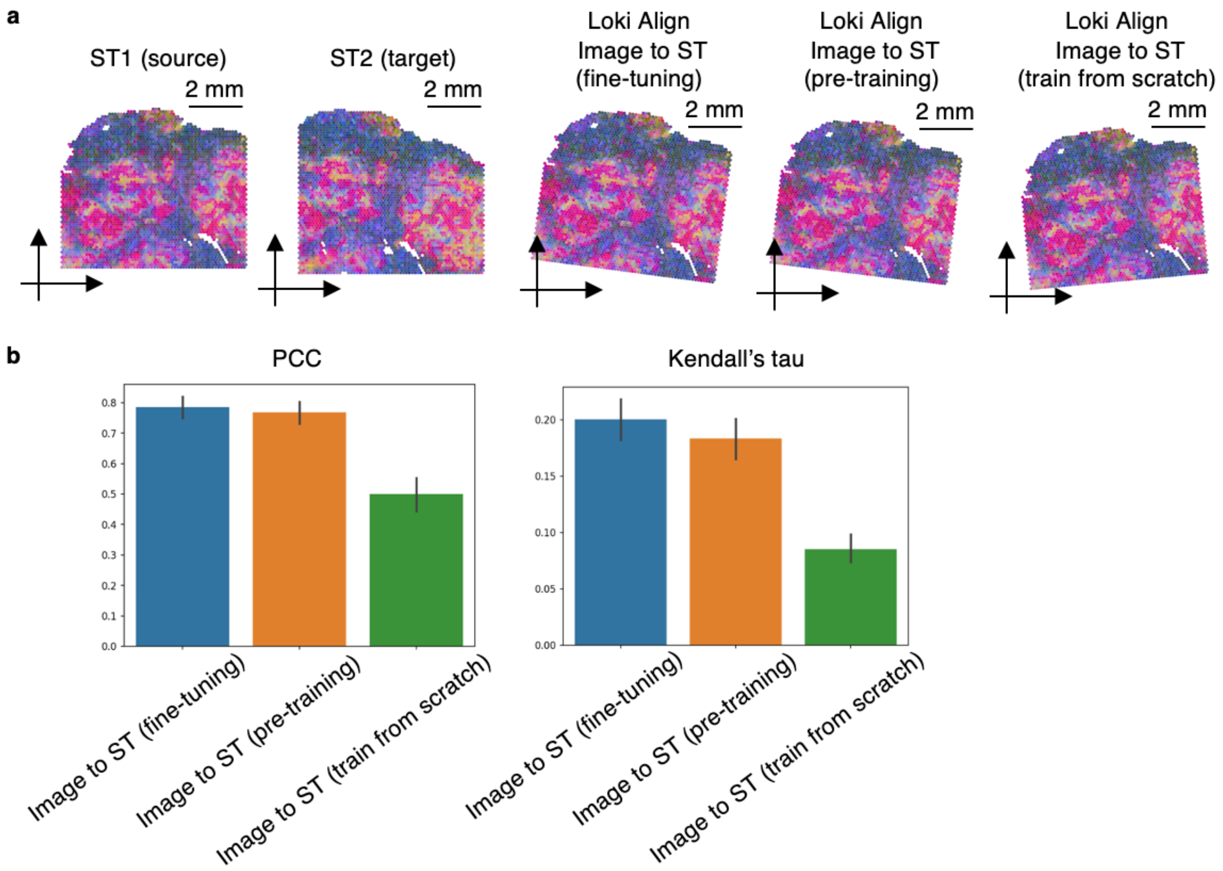

Supplementary Fig. 3: Tissue alignment of fine-tuning, pre-training, and train from scratch.

a, Tissue alignment results on 2 adjacent human ovarian carcinosarcoma samples using Loki Align Image-to-ST (fine-tuning, pre-training, and train from scratch). We colored the samples using the top three PCA components of OmiCLIP transcriptomic embeddings, mapped to red, green, blue color channels, respectively.

b, Comparison of tissue alignment performances, represented by the PCC and Kendall's tau coefficient of the highly expressed gene expression between target sample and source sample after alignment at the same location, using Loki Align Image-to-ST (fine-tuning, pre-training, and train from scratch). Bar plot shows the accuracy of alignment by Loki Align Image-to-ST (fine-tuning, pre-training, and train from scratch). Error bar is standard deviation with center measured by mean, n=147.

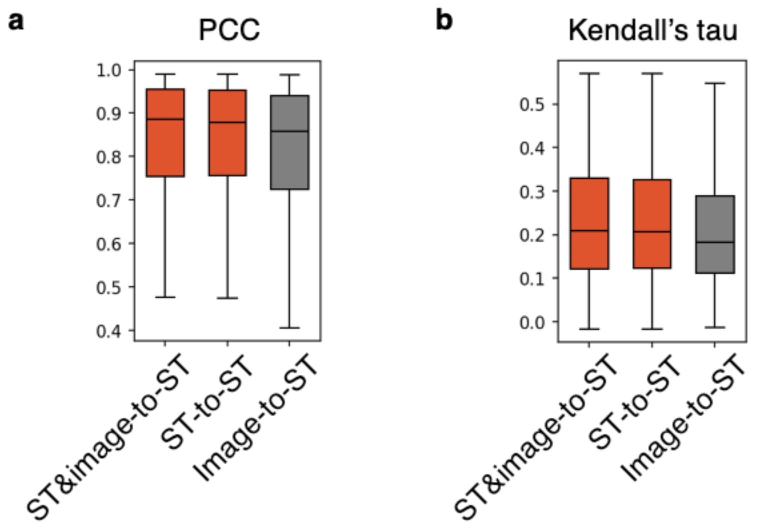

Supplementary Fig. 4: Tissue alignment using dual-modalities and single-modality.

a, Comparison of tissue alignment performances, represented by the PCC of the highly expressed gene expression between target sample and source sample after alignment at the same location, using Loki ST&image-to-ST, ST-to-ST, and Image-to-ST, respectively. In the box plots, the middle line represents the median, the box boundaries indicate the interquartile range, and the whiskers extend to data points within 1.5× the interquartile range, n=147.

b, Comparison of tissue alignment performances, represented by the Kendall’s tau coefficient of the highly expressed gene expression between target sample and source sample after alignment at the same location, using Loki ST&image-to-ST, ST-to-ST, and Image-to-ST, respectively. In the box plots, the middle line represents the median, the box boundaries indicate the interquartile range, and the whiskers extend to data points within 1.5× the interquartile range, n=147.

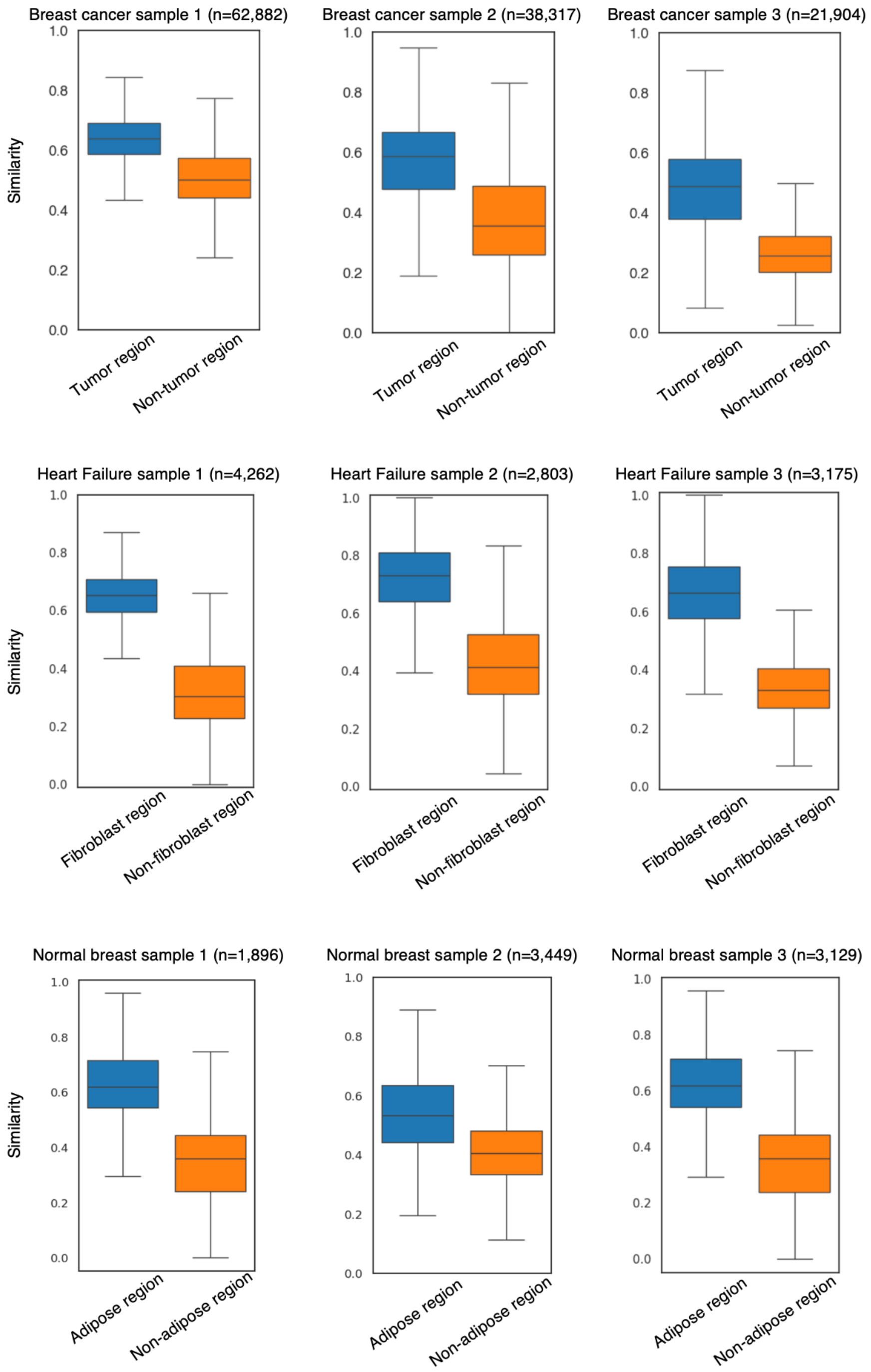

Supplementary Fig. 5: Cell type annotation.

Comparison of similarity difference on breast cancer with tumor bulk RNA-seq data, heart failure with fibroblast bulk RNA-seq data, and normal breast samples with tumor bulk RNA-seq data, within and out of the corresponding region annotated by pathology experts, respectively. In the box plots, the middle line represents the median, the box boundaries indicate the interquartile range, and the whiskers extend to data points within 1.5× the interquartile range.

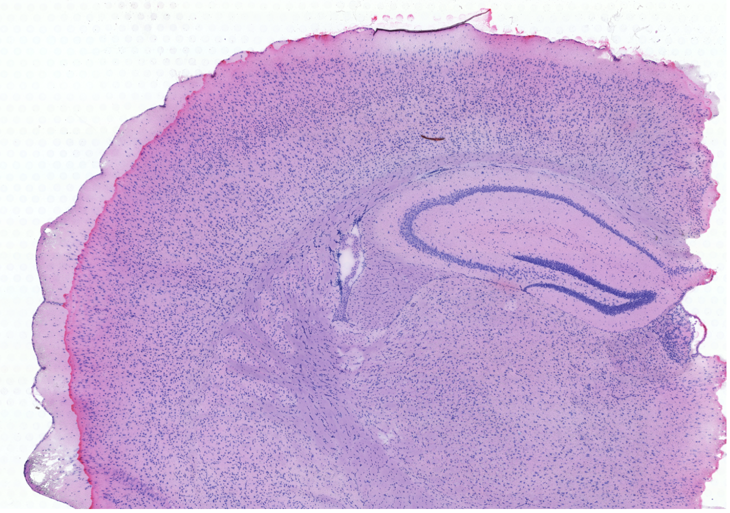

Supplementary Fig. 6: H&E image of the brain sample.

H&E image of the brain sample.

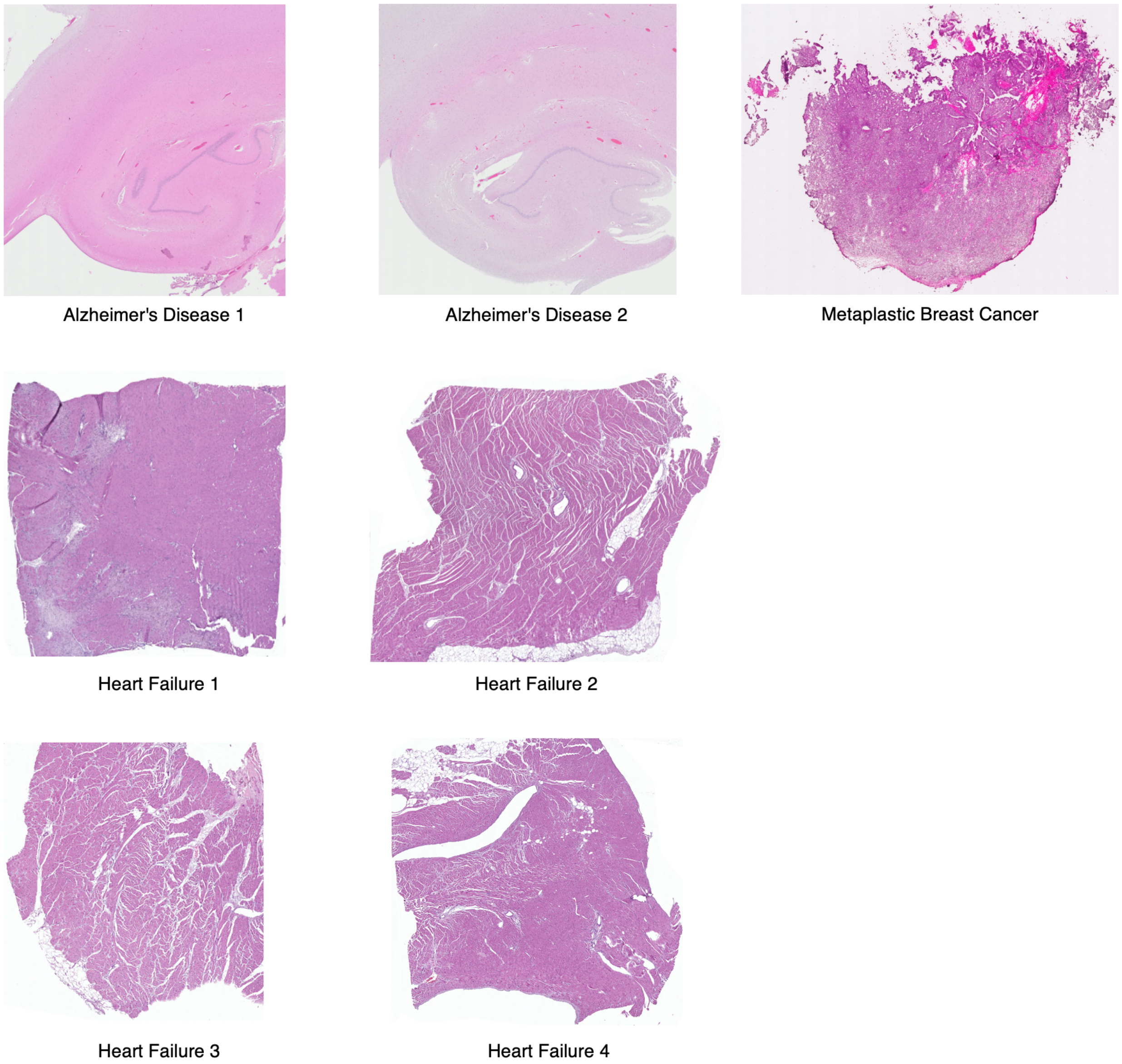

Supplementary Fig. 7: In-house patient samples.

HE images of our in-house patient samples with Alzheimer's disease, metaplastic breast cancer, and heart failure.

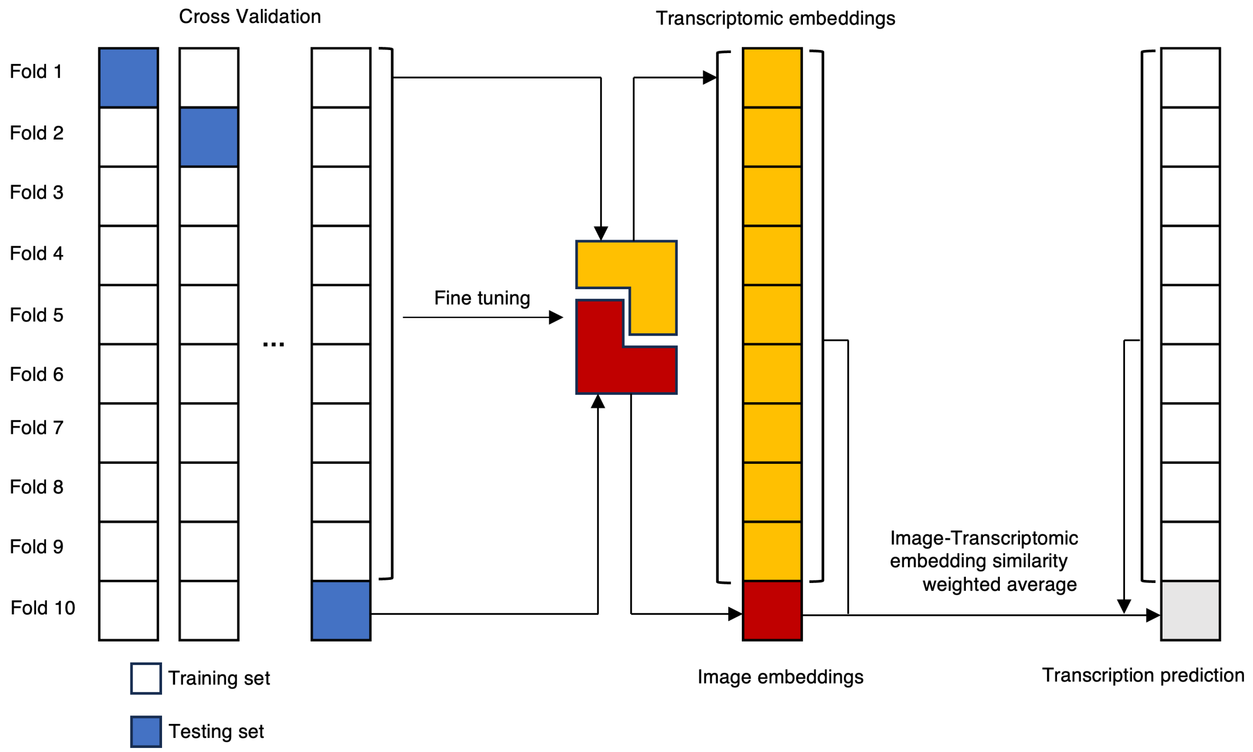

Supplementary Fig. 8: Loki ST gene expression prediction workflow.

Schematic illustration of Loki ST gene expression prediction using H&E images.

Supplementary Notes

Supplementary Note 1. Evaluating OmiCLIP's transcriptomic encoder on single-cell RNA-seq data

Our transcriptomics training data were collected from spot-level spatial transcriptomics, with each spot containing approximately 10-20 cells. Such training on spot-level data may lead to potential biases when encoding single-cell transcriptomics[1]. To evaluate the suitability of OmiCLIP's transcriptomic encoder on single-cell RNA sequencing (scRNA-seq) data, we tested whether its transcriptomic embeddings accurately capture cell heterogeneity by annotating cell types in scRNA-seq datasets.

We obtained two scRNA-seq atlas datasets collected from multiple patient samples, along with their corresponding cell-type annotations from external cancer studies as labels[2, 3]. Following standard quality control and preprocessing steps on the scRNA-seq data in SCANPY[4], gene symbols were ranked in descending order and converted into "sentences", excluding house-keeping genes. We then applied OmiCLIP's transcriptomic encoder to transform these gene name sentences into transcriptomic embeddings for cell type classification. Remarkably, OmiCLIP's transcriptomic embeddings achieved clear separation of cell types (Supplementary Fig. 1a).

For benchmarking, we compared OmiCLIP with OpenAI CLIP, a baseline model pretrained on general-domain text. We used identical procedures to convert the gene name sentences into OpenAI CLIP embeddings. Our analysis shows that OmiCLIP effectively clustered cells of same cell types from multi-sample scRNA-seq data. In contrast, OpenAI CLIP, pretrained on general-domain text, resulted in fragmented clusters of the same cell type and showed greater overlap among similar cell types (Supplementary Fig. 1b). This suggests that OmiCLIP's pretraining on domain-focused data, including gene names and histology image patches, enhances OmiCLIP's capability to group cells of the same type across samples more effectively, reflecting underlying biologically meaningful relationships inferred from transcriptomic and histological information.

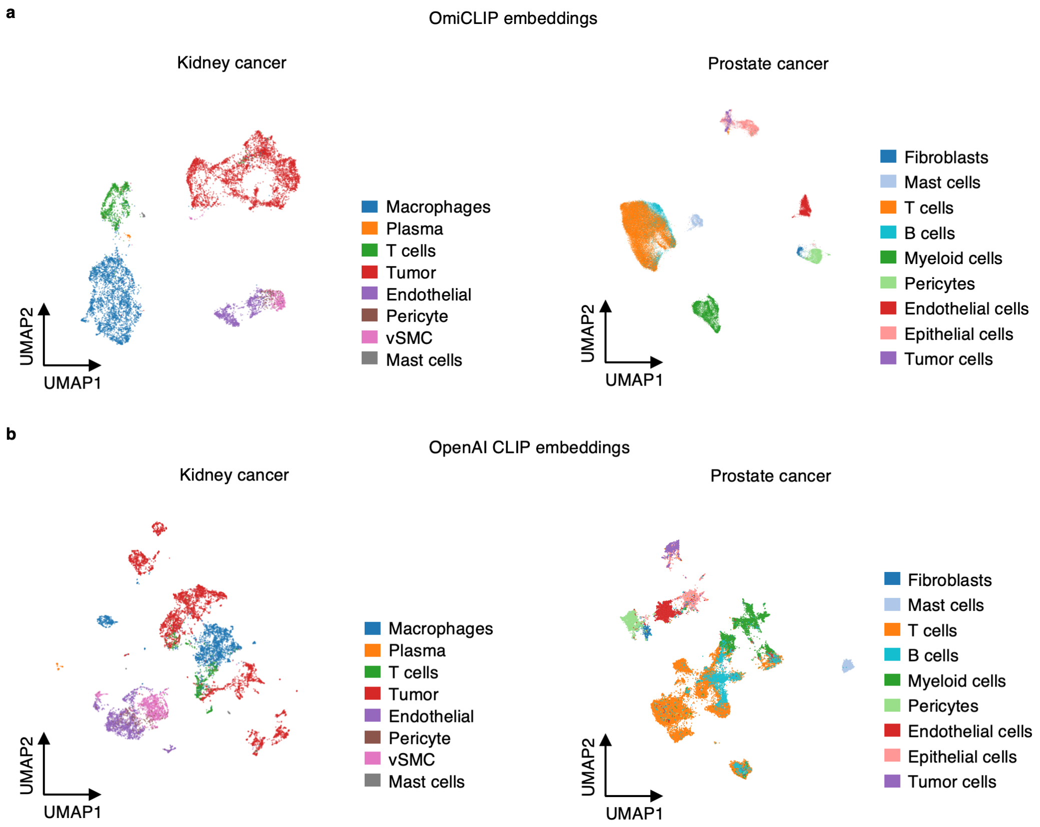

Supplementary Figure 1 UMAP visualizations of OmiCLIP/OpenAI CLIP transcriptomic embeddings of scRNA-seq data. a, UMAP representation of the OmiCLIP transcriptomic embeddings of single cells in kidney cancer and prostate cancer samples. Each dot represents a cell and the colors mark the cell type information from original study. **b,** Same as in (**a**) with OpenAI CLIP-based embeddings. Note for the prostate cancer OpenAI CLIP embeddings, tumor cells ('invisible') totally overlap with the epithelial cells.

In Loki platform, we employed the Gaussian Naive Bayes (Gaussian NB) algorithm (implemented via Scikit-Learn's GaussianNB module) for classification on the transcriptomic embeddings from our pretrained encoder. In the few-shot setting, we randomly sampled a small fraction of labeled examples per class for training, ranging from 0.1%, 0.5%, 1%, 2%, 3%, up to the maximum number of labeled examples available for a given class. The remainder of the data was used for validation. Each experiment was repeated 10 times with different random seeds to account for dataset variability. The performance of the classification was evaluated using the weighted F1 score.

OmiCLIP achieved high precision for most cell types, with the exception of particularly challenging classes, such as normal epithelial versus tumor epithelial cells (Supplementary Fig. 2a-b). In kidney cancer datasets, Loki achieved a median F1 score of 0.89 with only 0.5% of training labels per class, surpassing OpenAI CLIP (0.81) and outperforming conventional methods like SingleR[5] (0.50), Celltypist[6] (0.58), and scType[7] (0.32). Likewise, in prostate cancer samples, Loki achieved a median F1 score of 0.82 with only 0.1% of training labels per class (Supplementary Fig. 2c). Overall, OmiCLIP's transcriptomic embeddings accurately annotated cell types with as few as 0.5% labeled training data and consistently outperformed the OpenAI CLIP text embeddings for all sizes of the training sets in the two datasets. These results

indicated that OmiCLIP's transcriptomic encoder effectively captures the heterogeneity of single-cell transcriptomics.

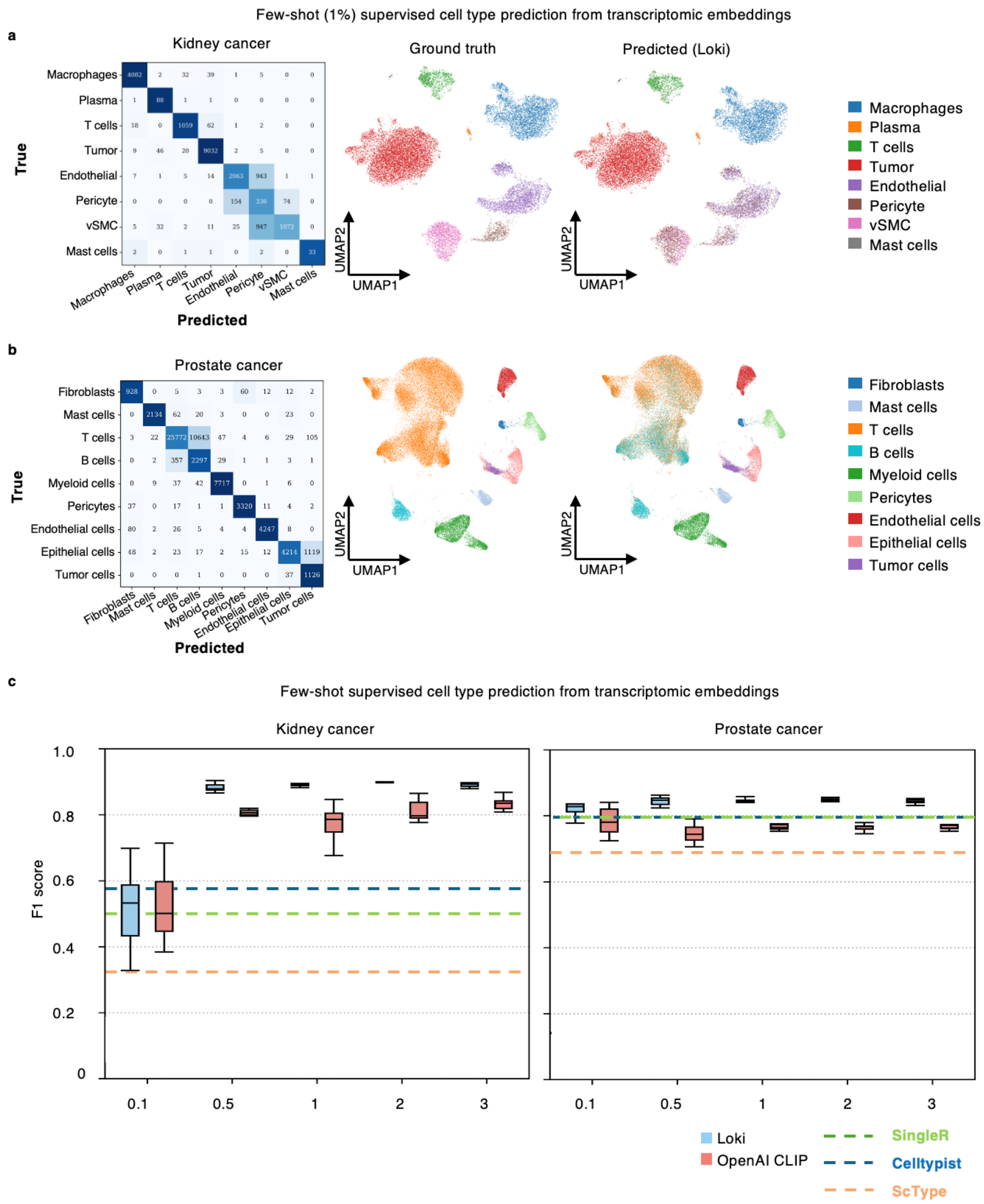

Supplementary Figure 2 Few-shot cell type annotation. a, Confusion matrix between predicted and actual cell types of the kidney cancer dataset. The matrix is colored from light to dark blue by the precision values. The projection of the actual and predicted cell type labels to the original UMAP embedding for the kidney cancer dataset. 1% randomly sampled cells were used for training and the rest were plotted on the UMAP embedding. **b**, same as in (a) for the prostate cancer dataset. **c,** Performance of cell type annotation in four scRNA-seq datasets measured by weighted F1 score. In the box plots, the middle line represents the median, the box boundaries indicate the interquartile range, and the whiskers extend to data points within 1.5× the interquartile range. The dashed lines represent the performance of conventional methods.

Supplementary Note 2. Cancer patient risk stratification in the TCGA dataset using OmiCLIP embeddings

Previous studies have shown that diagnostic WSI and RNA-seq can be integrated using deep learning for cancer prognosis via a late fusion model[8, 9]. Using The Cancer Genome Atlas (TCGA) patient data, we further tested the capability of OmiCLIP to bridge bulk RNA-seq data and paired WSI for leveraging patient risk stratification.

2.1 Biological relevance of OmiCLIP's transcriptomic embeddings in the TCGA bulk RNA-seq dataset

By converting RNA-seq expression data to gene name sentences, OmiCLIP's transcriptomic embeddings effectively separated patients of different cancer types (Supplementary Fig. 3a), achieving comparable performance of the original bulk RNA-seq expression data (Supplementary Fig. 3b).

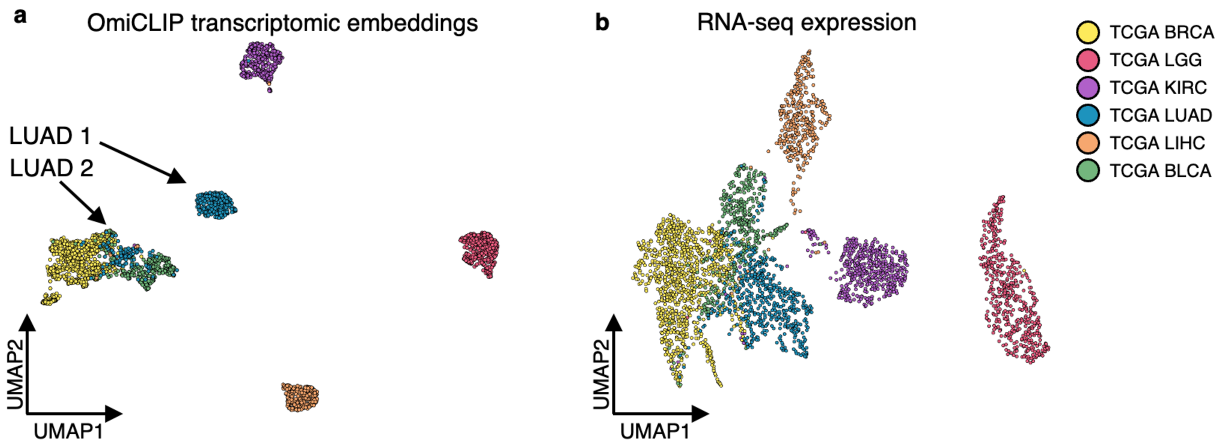

Supplementary Figure 3 OmiCLIP transcriptomic embeddings distinguish TCGA cancer types. a, UMAP representation of the OmiCLIP transcriptomic embeddings, where each dot represents a patient case. The OmiCLIP transcriptomic embeddings visually divide the TCGA LUAD patients into two groups: LUAD 1 and LUAD 2. **b**, UMAP representation based on TCGA RNA abundances.

We further investigated OmiCLIP's transcriptomic encoder on the TCGA bulk RNA-seq data. OmiCLIP's transcriptomic embeddings divided TCGA Lung Adenocarcinoma (LUAD) patients into two distinct groups (Supplementary Fig. 4a) as LUAD 1 and LUAD 2. To investigate the clinical insight added by this transcriptomic embedding, we performed Kaplan-Meier survival analysis on these two groups of patients. The results showed a significant difference in overall survival between the two groups (Supplementary Fig. 4b), indicating the prognostic relevance of OmiCLIP's transcriptomic encoder. To further investigate the underlying relevance of the OmiCLIP transcriptomic embeddings, we split the larger LUAD group into LUAD 1 – Prox (50%) and LUAD 1 – Dist (50%), which were close to and distant from the LUAD 2 group in OmiCLIP embedding space, respectively (Supplementary Fig. 4c). Kaplan-Meier survival analysis revealed a more significant difference in overall survival between the LUAD 1 – Dist and LUAD

2 groups, with a p-value of 7 x 10^{-4} (Supplementary Fig. 4d). This analysis indicated OmiCLIP's capability to identify patient clusters with clinical relevance. This capability potentially arises from incorporation of the biological knowledge derived from the literature: we initialized OmiCLIP's transcriptomic encoder using the pretraining weights of a Bidirectional Encoder Representations from Transformers (BERT) language model, thereby leveraging the power of natural language processing (NLP) models to embed biological information into a high-dimensional space. Similar phenomenon has been reported by recent publications such as GenePT[10] and cell2sentence[11], supporting our approach of utilizing text-based embeddings to capture rich biological information efficiently.

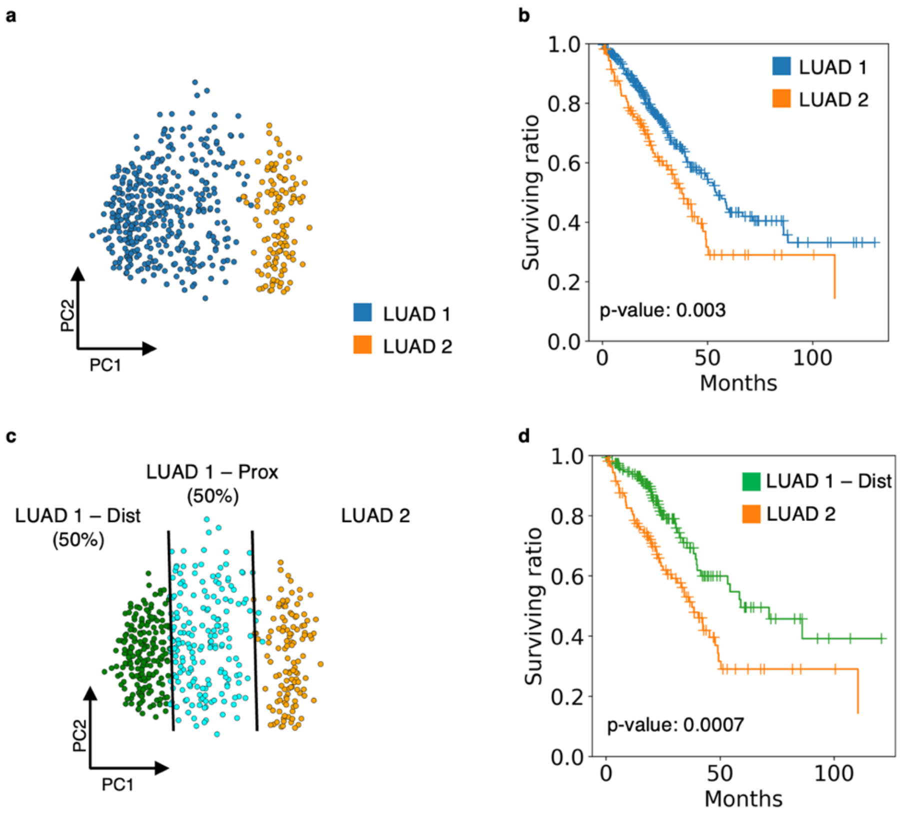

Supplementary Figure 4 OmiCLIP transcriptomic embeddings stratify patient groups on TCGA LUAD data. a, the PCA projections of the OmiCLIP transcriptomic embeddings of the TCGA LUAD patients colored by the clustering (LUAD 1 and LUAD 2). **b,** Kaplan-Meier curves for the LUAD 1 and LUAD 2 patient groups. The log rank test *p*-values are provided. **c,** the PCA projections of the OmiCLIP transcriptomic embeddings of the TCGA LUAD patients colored by LUAD 1 – Prox (in close proximity to LUAD 2), LUAD 1 – Dist (distant from LUAD 2), and LUAD 2. **d,** Kaplan-Meier curves for the LUAD 1 – Dist and LUAD 2 patient groups.

2.2 OmiCLIP's transcriptomics and image alignment achieves patient stratification in the TCGA dataset

Complementary to sequencing data, whole slide images (WSIs) provide phenotypic details, such as the morphology of normal tissue and tumor. Here we demonstrate that our multi-modal OmiCLIP model enables patient risk assessment across multiple cancer types without additional

training, using patients' H&E images and paired bulk RNA-seq data from the tumor biopsy. We hypothesized that integrating histology images could achieve patient stratification by incorporating morphological context, thus offering deeper insights into cancer risk and localized, region-specific explanations for tumor biology. To test this hypothesis, we employed OmiCLIP to calculate the similarity between individual tiles from WSIs and paired bulk RNA-seq data for each patient (Supplementary Fig. 5a). Since the transcriptome extracted from tumor biopsies reveals tumor-related features such as overexpression of *COL1A1*[12] and *ACTB*[13], the regions with high similarity highlight tumor regions-of-interest (ROIs). As demonstrated by the similarity heatmaps and example histology images of breast cancer samples in main Figure 4b, ROIs with higher similarity correspond to more malignant tissues (e.g. tumor cells). Using these cross-modal cosine similarity scores as weights, we computed a WSI-level image embedding by summing the tile embeddings—weighted by OmiCLIP's cross-modal similarity (Supplementary Fig. 5a). We then combined the multimodal OmiCLIP embeddings for each patient and fed them into a fully connected neural network for cancer risk estimation. The detailed methodology is described in the following.

We evaluated cancer risk at the patient level by merging diagnostic WSIs from the same patient into a single WSI, referred to as a patient WSI. For a given patient p, the top genes ranked by abundances were encoded by our pretrained text encoder into an L2-normalized transcriptomic embedding (T^p). Each WSI was first divided into tiles of 224 x 224 pixels using CLAM[14]. Tile embeddings (V_i^p for patient p and tile i) were computed using our pretrained image encoder. The cosine similarity (COS_i^p for patient p and tile i) between each tile embedding and the transcriptomic embedding were then computed and converted to probabilities using a softmax transformation,

$$COS_i^p = T^p \cdot V_i^p$$

$$P_i^p = \frac{\exp\,(COS_i^p)}{\sum_j \exp\,(COS_j^p)}$$

The tile probability P_i^p was used to aggerate the tile embeddings into a patient representation,

$$\tilde{V}^p = \sum_i V_i^p * P_i^p$$

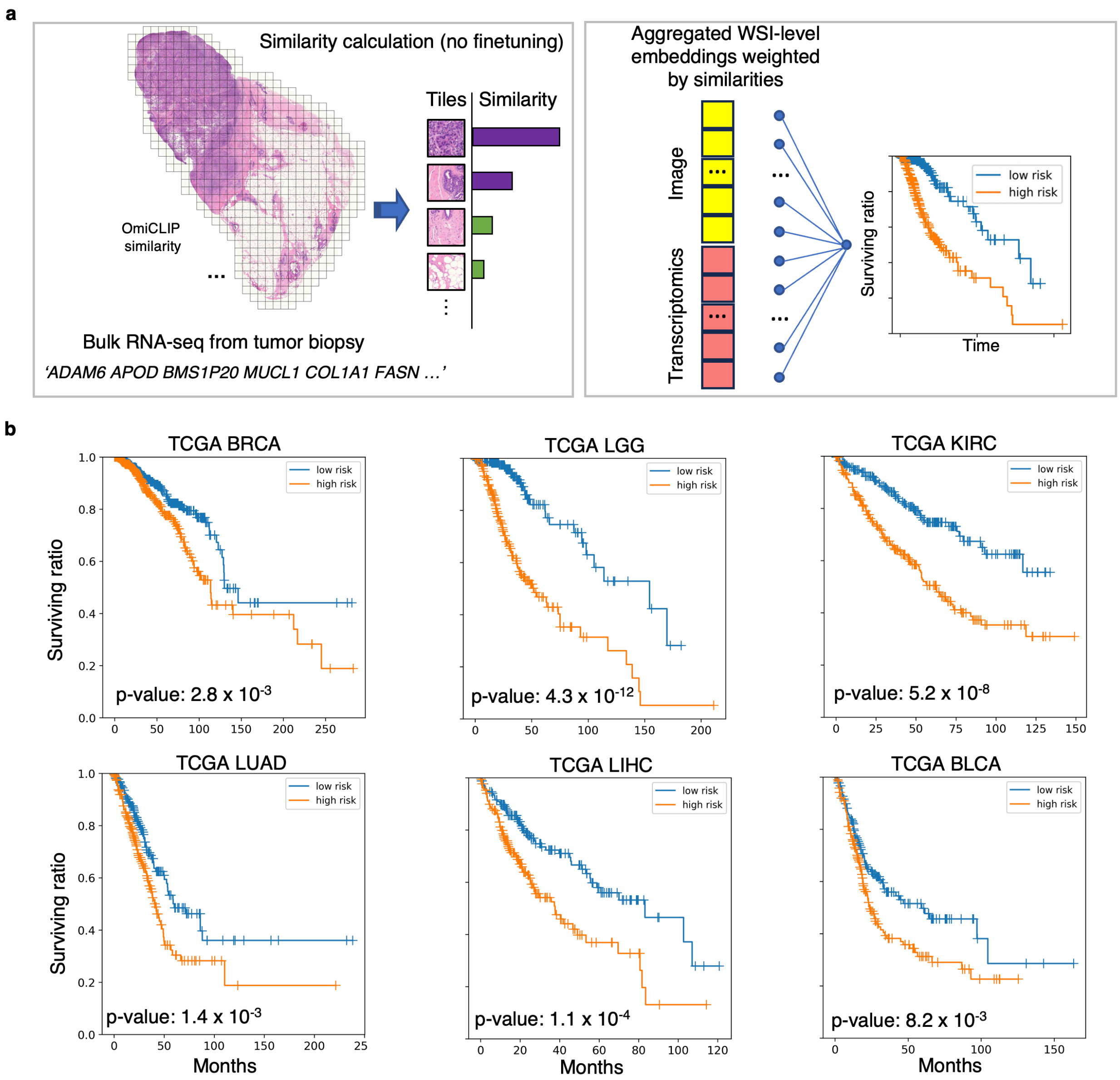

Supplementary Figure 5 Patient risk stratification in TCGA dataset. a, Schematic illustration of patient stratification from WSI and paired RNA-seq abundance with OmiCLIP paired image and transcriptomic embeddings. Each WSI (with tissue) is divided into tiles of size 224 × 224 pixels, and a weight is given to each patch according to its similarity to bulk RNA-seq data of the same patient's biopsy. The patch embeddings are summed to construct a WSI embedding weight by similarity. The multimodal patient-level embeddings are input to a simple neural network for cancer risk estimation. **b,** Kaplan-Meier curves for the high- and low-risk patient groups in six TCGA cancer datasets. The log rank test p-values are provided.

The patient level multi-modal embeddings (T^p and $\tilde{V}^p$) were then fed into a simple neural network for estimation of cancer risk. The network compromises two linear layers with ReLU activation functions to create a fused latent representation, and a subsequential linear classifier layer to output probabilities for high-risk prediction. The right-censored survival times were discretized into four non-overlapping, quantile-based bins. These bins were used as prediction

labels for training the model by maximizing a log likelihood function of the predicted survival bin index, following the methodologies from prior studies[8, 15]. For each cancer type, a model was trained for 20 epochs on the training set using the Adam optimizer, with a learning rate of $2×10^{-3}$ and a weight decay of $1×10^{-5}$. An L1-regularization with a coefficient of $1×10^{-5}$ was applied. Performance evaluation was conducted via five-fold validation, using Kaplan-Meier analysis for risk stratification, and the log rank test for assessing the statistical significance.

We evaluated the performance using five-fold cross-validation on TCGA data across six cancer types with most samples of paired WSI and RNA-seq data available, including Breast Invasive Carcinoma (BRCA), Low-Grade Glioblastoma (LGG), Kidney Renal Clear Cell Carcinoma (KIRC), Lung Adenocarcinoma (LUAD), Liver Hepatocellular Carcinoma (LIHC), and Bladder Urothelial Carcinoma (BLCA). We kept all the cases with both RNA-seq and diagnostic WSIs. Patients in each cancer type were grouped into lower and higher risk categories based on the 50th percentile of the predicted risk scores. A log-rank test was then applied to assess the statistical significance of the patient survival time in the two patient groups. We demonstrated that in all six cancer types, OmiCLIP's multimodal embeddings effectively stratified patient groups, revealing significant separation in the Kaplan-Meier curves (Supplementary Fig. 5b).

Altogether, through zero-shot capability of OmiCLIP pretraining that integrates local histopathology image regions and molecular features with spatial correspondences, we robustly characterized patient stratification and detected intra-tumoral heterogeneity. In contrast, weakly supervised deep learning techniques, which are widely used via a late-fusion approach[8], processing bulk molecular profile data and WSIs independently before combining them only at the decision stage, thereby potentially missing crucial interactions between the modalities. The pretrained OmiCLIP overcomes this limitation by learning a joint representation that captures complex interactions. This approach fuses the transcriptomics and H&E image earlier than when training the decision network. Despite not being specifically trained on TCGA, OmiCLIP effectively aligns images and bulk RNA-seq data, showing excellent agreement between the cosine similarity scores of WSI tiles and RNA-seq data from tumor biopsies. This facilitates early fusion for cancer risk estimation and demonstrates OmiCLIP's ability to generalize across various tasks and domains.

Supplementary Note 3. Downstream evaluation datasets

Tissue alignment: Simulated datasets were generated by perturbing real ST data and paired H&E images from 10 human tissue slices of diverse tissue and disease types including two breast cancer[16,17], one colorectal liver cancer[18], one liver cancer[19], one prostate cancer[20], one 10x Genomics prostate cancer, one 10x Genomics colon cancer, one embryonic lung[21], one normal small intestine[22], and one sleep apnea tonsil sample[23]. We simulated new ST experiments by perturbing both gene expression and spatial locations at different levels of noise. Gene expression perturbations involved adding pseudo counts drawn from a multinomial distribution, with the total counts per spot following a negative binomial distribution. Spatial locations perturbations involved cutting parts of the slice and rotating the coordinates. Low-noise perturbations had pseudo counts from 0.05 to 0.25, cut region from 3% to 10%, and rotations from 5 to 15 degrees. High-noise perturbations had pseudo counts from 0.25 to 0.5, cut region from 10% to 20%, and rotation degrees from 15 to 30. Paired H&E images were correspondingly cut and rotated. 10 simulated datasets were generated at different perturbation levels from each real datasets, totaling 200 datasets including 100 low-noise and 100 high-noise perturbations. Real-world data tests used a normal human small intestine dataset[22] including Visium ST data and paired H&E images of 8 adjacent tissue slices, a human ovarian carcinosarcoma dataset[24] including Visium ST data and paired H&E images of 2 adjacent tissue slices, and a human breast cancer dataset[25] including paired Visium and Xenium ST data.

Tissue annotation: Bulk RNA-seq data based annotation used 3 normal human breast and 3 human heart failure histology images[26,27], and 3 breast cancer patient histology images TCGA. Pathology experts annotated different tissue regions. Bulk RNA-seq datasets including 663 human adipose and 504 human fibroblast samples from the Genotype-Tissue Expression (GTEx) Portal and 3 paired tumor biopsies samples from TCGA. Gene expressions were averaged for adipose and fibroblast samples. For the tissue annotation task using marker genes, we include 4 external validation datasets: CRC7K, WSSS4LUAD, LC25000, and PatchCamelyon. CRC7K consisted of 7,180 image patches of 224×224 pixels at 0.5 microns per pixel (MPP) from 50 patients with colorectal adenocarcinoma. All images were categorized into one of the following nine classes: adipose, background, debris, lymphocytes, mucus, smooth muscle, normal colon mucosa, cancer-associated stroma, or colorectal adenocarcinoma epithelium. We removed background images and that resulted in 6,333 image patches with 8 different tissue types. As the MPP value is about 2 times our training data, we cropped the images into four non-overlapping tiles. The image embeddings encoded by OmiCLIP were the average of these four tiles. WSSS4LUAD consisted of 10,091 LUAD image patches of around 200–500 pixels from 63 WSIs. All images were categorized as either tumor or normal, depending on the presence of tumor tissue. This resulted in 6,579 tumor images and 3,512 normal images. LC25000 consisted of 25,000 lung and colon image patches of 768×768 pixels. We used the subset of all lung tissues with 5,000 images of lung adenocarcinomas, 5,000 images of lung squamous cell carcinomas, and 5,000 images of benign lung tissues. All images were categorized as either cancer or benign. This resulted in 10,000 cancer images and 5,000 benign images. PatchCamelyon consisted of 327,680 image patches of 96 x 96 pixels from

lymph node sections. All images were categorized as either tumor or normal depending on the presence of metastatic tissue. We used the validation set with 32,768 image patches.

Cell type decomposition: We downloaded a human colorectal cancer dataset[28] including Visium, Visium-HD, and scRNA-seq data of serial slices. We used the Visium-HD 8-mm bin data as the ground truth and created pseudo-Visium spots in the Visium-HD capture area, mirroring the Visium spot configuration that each spot is 55 μm in diameter with a 100 μm center-to-center distance between spots. Different tissue regions are annotated by pathology experts. We also collected one in-house triple-negative breast cancer patient-derived xenograft. The sample was snap freeze using 10x Genomics protocol # CG000663. Frozen OCT tumors were sectioned using cryostat as per 10x Genomics protocol # CG000579 (Rev E) and mounted directly on the Xenium slide. Next, sections on Xenium slides were processed as per 10x Genomics protocol # CG000613 (Rev B). For the probes, we used a 10x pre-designed human breast panel that has 280 genes on the panel. Slides were profiled using Xenium Analyzer (10x Genomics). Following analysis, Xenium slides were processed for Hematoxylin & Eosin (H&E) staining using 10x Genomics protocol # CG000613. H&E slides were imagined using Akoya Bioscience Phenocycler Fusion. We annotated the tumor epithelial cells, immune cells, and stroma cells based on marker genes respectively. We then created pseudo Visium spots based on the Xenium data resulting in 3,402 spots, mirroring the Visium spot configuration that each spot is 55 μm in diameter with a 100 μm center to center distance between spots. To decompose this TNBC sample, we downloaded an external scRNA-seq of TNBC as a reference[29]. We also downloaded an external mouse brain dataset[30] including Visium data of serial slices, and used a reference mouse brain scRNA-seq dataset[31] from the Allen Institute.

H&E image-to-ST retrieval: Our in-house heart failure patient tissues were obtained at Houston Methodist Hospital under an approved IRB protocol: Pro 00006097 congestive heart failure with standard protocol for H&E staining. Our in-house paraffin-embedded Alzheimer's disease patient samples were stained following standard protocol for H&E staining. Our in-house metaplastic breast cancer and triple-negative breast cancer patient-derived xenografts were collected and snap-frozen using 10x Genomics protocol # CG000663, and H&E stained using 10x Genomics protocol # CG000613. The validation datasets including brain, heart, kidney, and breast samples, and the test dataset including desmoplastic small round cell tumor, colorectal cancer, vascular, and colon samples from 4 studies (Supplementary Table 4).

ST gene expression prediction: We used a normal human heart sample dataset[27] of paired ST data and H&E images including 39 samples.

References

1. Zhou, Z., Zhong, Y., Zhang, Z. & Ren, X. Spatial transcriptomics deconvolution at single-cell resolution using Redeconve. Nature Communications 14, 7930 (2023).
2. Hirz, T. et al. Dissecting the immune suppressive human prostate tumor microenvironment via integrated single-cell and spatial transcriptomic analyses. Nat Commun 14, 663 (2023).
3. Zhang, Y. et al. Single-cell analyses of renal cell cancers reveal insights into tumor microenvironment, cell of origin, and therapy response. Proc Natl Acad Sci U S A 118 (2021).
4. Wolf, F.A., Angerer, P. & Theis, F.J. SCANPY: large-scale single-cell gene expression data analysis. Genome Biol 19, 15 (2018).
5. Aran, D. et al. Reference-based analysis of lung single-cell sequencing reveals a transitional profibrotic macrophage. Nat Immunol 20, 163-172 (2019).
6. Dominguez Conde, C. et al. Cross-tissue immune cell analysis reveals tissue-specific features in humans. Science 376, eabl5197 (2022).
7. Ianevski, A., Giri, A.K. & Aittokallio, T. Fully-automated and ultra-fast cell-type identification using specific marker combinations from single-cell transcriptomic data. Nat Commun 13, 1246 (2022).
8. Chen, R.J. et al. Pan-cancer integrative histology-genomic analysis via multimodal deep learning. Cancer Cell 40, 865-878. e866 (2022).
9. Chen, R.J. et al. in Proceedings of the IEEE/CVF International Conference on Computer Vision 4015-4025 (2021).
10. Chen, Y. & Zou, J. Simple and effective embedding model for single-cell biology built from ChatGPT. Nat Biomed Eng (2024).
11. Levine, D. et al. Cell2sentence: Teaching large language models the language of biology. BioRxiv, 2023.2009. 2011.557287 (2023).
12. Hayashi, M. et al. Identification of the collagen type 1 alpha 1 gene (COL1A1) as a candidate survival-related factor associated with hepatocellular carcinoma. BMC cancer 14, 1-10 (2014).
13. Gu, Y. et al. A pan-cancer analysis of the prognostic and immunological role of β-actin (ACTB) in human cancers. Bioengineered 12, 6166-6185 (2021).
14. Lu, M.Y. et al. Data-efficient and weakly supervised computational pathology on whole-slide images. Nature biomedical engineering 5, 555-570 (2021).
15. Song, A.H. et al. Analysis of 3D pathology samples using weakly supervised AI. Cell 187, 2502-2520. e2517 (2024).
16. He, S., et al. Starfysh reveals heterogeneous spatial dynamics in the breast tumor microenvironment. bioRxiv, 2022.2011. 2021.517420 (2022).
17. Barkley, D., et al. Cancer cell states recur across tumor types and form specific interactions with the tumor microenvironment. Nature genetics 54, 1192-1201 (2022).
18. Garbarino, O., et al. Spatial resolution of cellular senescence dynamics in human colorectal liver metastasis. Aging Cell 22, e13853 (2023).
19. Wu, R., et al. Comprehensive analysis of spatial architecture in primary liver cancer. Science Advances 7, eabg3750 (2021).
20. Figiel, S., et al. Spatial transcriptomic analysis of virtual prostate biopsy reveals confounding effect of tissue heterogeneity on genomic signatures. Molecular Cancer 22, 162 (2023).
21. Sountoulidis, A., et al. A topographic atlas defines developmental origins of cell heterogeneity in the human embryonic lung. Nature Cell Biology 25, 351-365 (2023).

22. Mirzazadeh, R., et al. Spatially resolved transcriptomic profiling of degraded and challenging fresh frozen samples. Nature Communications 14, 509 (2023).
23. Engblom, C., et al. Spatial transcriptomics of B cell and T cell receptors reveals lymphocyte clonal dynamics. Science 382, eadf8486 (2023).
24. Kumar, T., et al. A spatially resolved single-cell genomic atlas of the adult human breast. Nature 620, 181-191 (2023).
25. Villacampa, E.G., et al. Genome-wide spatial expression profiling in formalin-fixed tissues. Cell Genomics 1(2021).
26. Kuppe, C., et al. Spatial multi-omic map of human myocardial infarction. Nature 608, 766-777 (2022).
27. Kanemaru, K., et al. Spatially resolved multiomics of human cardiac niches. Nature 619, 801-810 (2023).
28. Oliveira, M.F., et al. Characterization of immune cell populations in the tumor microenvironment of colorectal cancer using high definition spatial profiling. bioRxiv, 2024.2006. 2004.597233 (2024).
29. Karaayvaz, M., et al. Unravelling subclonal heterogeneity and aggressive disease states in TNBC through single-cell RNA-seq. Nature communications 9, 3588 (2018).
30. Kleshchevnikov, V., et al. Cell2location maps fine-grained cell types in spatial transcriptomics. Nature biotechnology 40, 661-671 (2022).
31. Tasic, B., et al. Shared and distinct transcriptomic cell types across neocortical areas. Nature 563, 72-78 (2018).
