# Supplementary material for: A visual–omics foundation model to bridge histopathology with spatial transcriptomics
Source: Nat Methods. 2025 May 29;22(7):1568–82. doi: 10.1038/s41592-025-02707-1 (PMC12240810; doi:10.1038/s41592-025-02707-1)
Supplement: Supplementary file 1 [file 41592_2025_2707_MOESM2_ESM.pdf]

Corresponding author(s): Guangyu Wang

Last updated by author(s): Mar 27, 2025

Reporting Summary

Nature Portfolio wishes to improve the reproducibility of the work that we publish. This form provides structure for consistency and transparency in reporting. For further information on Nature Portfolio policies, see our Editorial Policies and the Editorial Policy Checklist.

Statistics

For all statistical analyses, confirm that the following items are present in the figure legend, table legend, main text, or Methods section.

n/a	Confirmed	
☐	☒	The exact sample size (*n*) for each experimental group/condition, given as a discrete number and unit of measurement
☒	☐	A statement on whether measurements were taken from distinct samples or whether the same sample was measured repeatedly
☐	☒	The statistical test(s) used AND whether they are one- or two-sided<br>*Only common tests should be described solely by name; describe more complex techniques in the Methods section.*
☒	☐	A description of all covariates tested
☒	☐	A description of any assumptions or corrections, such as tests of normality and adjustment for multiple comparisons
☐	☒	A full description of the statistical parameters including central tendency (e.g. means) or other basic estimates (e.g. regression coefficient) AND variation (e.g. standard deviation) or associated estimates of uncertainty (e.g. confidence intervals)
☐	☒	For null hypothesis testing, the test statistic (e.g. *F*, *t*, *r*) with confidence intervals, effect sizes, degrees of freedom and *P* value noted<br>*Give P values as exact values whenever suitable.*
☒	☐	For Bayesian analysis, information on the choice of priors and Markov chain Monte Carlo settings
☒	☐	For hierarchical and complex designs, identification of the appropriate level for tests and full reporting of outcomes
☐	☒	Estimates of effect sizes (e.g. Cohen's *d*, Pearson's *r*), indicating how they were calculated

Our web collection on statistics for biologists contains articles on many of the points above.

Software and code

Policy information about availability of computer code

Data collection	The pre-training data were processed in Python v.3.9 by pandas v.2.2.3; numpy v.1.25.0; and scanpy v.1.10.3.
Data analysis	All experiments were run in Python v.3.9. Detailed software versions are CUDA v.12.2; torch v.2.3.1; torchvision v.0.18.1; scipy v.1.13.1; pillow v.10.4.0; scikit-learn v.1.5.2; pandas v.2.2.3; numpy v.1.25.0; and scanpy v.1.10.3.

For manuscripts utilizing custom algorithms or software that are central to the research but not yet described in published literature, software must be made available to editors and reviewers. We strongly encourage code deposition in a community repository (e.g. GitHub). See the Nature Portfolio guidelines for submitting code & software for further information.

Data

Policy information about availability of data

All manuscripts must include a data availability statement. This statement should provide the following information, where applicable:

- Accession codes, unique identifiers, or web links for publicly available datasets
- A description of any restrictions on data availability
- For clinical datasets or third party data, please ensure that the statement adheres to our policy

The normal human small intestine dataset used for the tissue alignment task can be found at: https://doi.org/10.1038/s41467-023-36071-5. The human ovarian carcinosarcoma dataset used for the tissue alignment task can be found at: https://doi.org/10.1016/j.xgen.2021.100065. The human breast cancer dataset used for the tissue alignment task can be found at: https://doi.org/10.1038/s41467-023-43458-x. The human colorectal cancer dataset including Visium, Visium-HD, and

scRNA-seq data of serial slices used for cell type decomposition task can be found at: https://doi.org/10.1101/2024.06.04.597233. The TNBC scRNA-seq data used for the cell type decomposition task can be found at: https://doi.org/10.1038/s41467-018-06052-0. The TNBC Xenium data generated in this study have been deposited in the GEO database under accession code GSE293199. The brain dataset including Visium data of serial slices used for cell type decomposition task can be found at: https://www.nature.com/articles/s41587-021-01139-4. The brain scRNA-seq dataset used for cell type decomposition task can be found at: https://www.nature.com/articles/s41586-018-0654-5. The histology images of the heart failure patient dataset used for the tissue annotation task can be found at: https://doi.org/10.1038/s41586-022-05060-x. The histology images of the normal human breast dataset used for the tissue annotation task can be found at: https://doi.org/10.1038/s41586-023-06252-9. The histology images of the TCGA BRCA dataset used for the tissue annotation task are available from the NIH Genomic Data Commons (http://portal.gdc.cancer.gov). The bulk RNA-seq data used for tissue annotation task are available from the Genotype-Tissue Expression (GTEx) Portal (https://gtexportal.org/home/) and TCGA (http://portal.gdc.cancer.gov). CRC7k image patches data and labels can be found at http://zenodo.org/record/1214456. WSSS4LUAD image patches and labels can be found at http://wsss4luad.grand-challenge.org. LC25000 image patches and labels can be found at https://github.com/tampapath/lung_colon_image_set. PatchCamelyon image patches and labels can be found at https://patchcamelyon.grand-challenge.org. The validation and test datasets used for the image–transcriptomics retrieval task can be found in Supplementary Table 4. The normal human heart samples used for the ST gene expression prediction task can be found at https://doi.org/10.1038/s41586-023-06311-1. ST bank database is available at GitHub: https://github.com/GuangyuWangLab2021/Loki.

Human research participants

Policy information about studies involving human research participants and Sex and Gender in Research.

Reporting on sex and gender	No sex- or gender-based analysis in this study as all the data consisted of de-identified samples.
Population characteristics	N/A
Recruitment	N/A
Ethics oversight	N/A

Note that full information on the approval of the study protocol must also be provided in the manuscript.

Field-specific reporting

Please select the one below that is the best fit for your research. If you are not sure, read the appropriate sections before making your selection.

☒ Life sciences ☐ Behavioural & social sciences ☐ Ecological, evolutionary & environmental sciences

For a reference copy of the document with all sections, see nature.com/documents/nr-reporting-summary-flat.pdf

Life sciences study design

All studies must disclose on these points even when the disclosure is negative.

Sample size	2,185,571 pathology images paired with transcriptom were used in this study to ensure an adequate representation of the data under investigation. No sample size calculations were performed. Sample size was determined by data acquisition pipeline.
Data exclusions	A rigorous quality control was applied to remove duplicates, exclude low-resolution H&E images (<2,000 × 2,000 pixels), and filter out contaminated, extremely low-quality, or damaged cells, retaining only those with over 200 expressed genes.
Replication	Replication was successful for all experiments in this study.
Randomization	Randomization was not applicable to this study as there was no comparison groups.
Blinding	Blinding was not applicable to this study as all the data consisted of de-identified samples.

Reporting for specific materials, systems and methods

We require information from authors about some types of materials, experimental systems and methods used in many studies. Here, indicate whether each material, system or method listed is relevant to your study. If you are not sure if a list item applies to your research, read the appropriate section before selecting a response.

Materials & experimental systems

n/a	Involved in the study
☒	☐ Antibodies
☒	☐ Eukaryotic cell lines
☒	☐ Palaeontology and archaeology
☒	☐ Animals and other organisms
☒	☐ Clinical data
☒	☐ Dual use research of concern

Methods

n/a	Involved in the study
☒	☐ ChIP-seq
☒	☐ Flow cytometry
☒	☐ MRI-based neuroimaging
